# Supplementary material for: Socio-Economic Factors Associated with Ethnic Disparities in SARS-CoV-2 Infection and Hospitalization
Source: Int J Environ Res Public Health. 2023 Aug 4;20(15):6521. doi: 10.3390/ijerph20156521 (PMC10418672; doi:10.3390/ijerph20156521)
Supplement: Supplementary file 1 [file ijerph-20-06521-s001.zip › ijerph-2473947-supplementary.pdf]

Table S1. Standardized Cumulative Incidence Rate (SCIR) and Standardized Cumulative Hospitalization Rate (SCHR) for 100,000 inhabitants (European population 2013)

VARIABLES	SCIR			SCHR		
	Italian	Non-Italian	Total	Italian	Non-Italian	Total
**Overall**	6539	5753	-	353	421	-
**Sex**						
Male	6630	5962	-	449	492	-
Females	6469	5593	-	269	377	-
**HDI**						
Very high	-	-	3136	-	-	160
Italy	-	-	6539	-	-	353
High	-	-	5803	-	-	419
Medium	-	-	6807	-	-	520
Low	-	-	5967	-	-	874
**Deprivation Index**						
Quintile 1	-	-	5917	-	-	353
Quintile 2	-	-	5997	-	-	312
Quintile 3	-	-	6963	-	-	413
Quintile 4	-	-	6933	-	-	412
Quintile 5	-	-	6710	-	-	387
**Urban-Rural Index**						
A-pole	-	-	6783	-	-	398
A1-urban	-	-	7946	-	-	476
A2-rural	-	-	6828	-	-	363
B2-rural	-	-	5883	-	-	325
C2-rural	-	-	5543	-	-	299
**Country**						
Romania	-	-	4545	-	-	239
Albania	-	-	7870	-	-	514
Morocco	-	-	7307	-	-	479
Ukraine	-	-	5329	-	-	354
North Macedonia	-	-	4699	-	-	290
Ecuador	-	-	14327	-	-	1204
Nigeria	-	-	7098	-	-	1799
Moldova (Republic of)	-	-	4790	-	-	319
China	-	-	3665	-	-	1077
Philippines	-	-	3199	-	-	395
**Continents**						
America	-	-	9373	-	-	681
Africa	-	-	6462	-	-	589
Europa	-	-	6446	-	-	352
Asia	-	-	3749	-	-	428
Oceania	-	-	2545	-	-	0
